# Expression of HERV-H/W env epitopes on PBMCs from MS patients with active disease

**DOI:** 10.1186/1742-4690-8-S1-A210

**Published:** 2011-06-06

**Authors:** Tomasz Brudek, Tove Christensen, Thor Petersen, Anné Møller-Larsen

**Affiliations:** 1 Department of Medical Microbiology and Immunology, Aarhus University, Aarhus C, DK-8000, Denmark; 2 Research Laboratory for Stereology and Neuroscience, Bispebjerg University Hospital, København NV, DK-2400, Denmark; 3 Department of Neurology, Aarhus University Hospital, Aarhus C, DK-8000, Denmark

## Background

The demyelinating disease Multiple Sclerosis (MS) is assumed to be caused by a malfunction of the immune system, maybe due to exposure of genetically susceptible individuals to unknown environmental agent(s) - possibly virus. Our working hypothesis is that these viruses could be endogenous retroviruses, activated by other infectious agents, presumably from the herpes virus group with EBV as the prime candidate. Previously retroviral activity has been monitored by PERT assays. Assays such as flow cytometry enables detection of possible expression of viral epitopes on the surface of PBMCs from MS-patients, and confocal microscopy can show the cellular location of these epitopes.

## Materials and methods

Polyclonal rabbit antibodies against HERV-H/W Env SU- and TM-regions were used in flow cytometry to detect cell-membrane expression of these epitopes on PBMCs from MS patients in different disease states compared with healthy individuals, and patients with other neurological diseases. Monoclonal antibodies against CD-epitopes were used to quantitate the different PBMCs expressing the HERV-epitopes. The rabbit antibodies were also used in labeling of long-term, spontaneously growing, lymphoblastoid cell-cultures from MS patients to localize viral epitopes on the cell surfaces.

## Results

The flow cytometric analyses detect increased quantities of HERV-H/W Env epitopes on B-cells and monocytes together with increased numbers of B-cells in patients with active MS. Confocal microscopy show expression of viral epitopes on the surface of the lymphoid cell cultures.

## Conclusions

The findings demonstrate higher expression of HERV-H/W Env epitopes on the surface of some types of PBMCs from patients with active MS.

